# Evolution of Microstructures and Mechanical Properties with Tempering Temperature in a Novel Synergistic Precipitation Strengthening Ultra-High Strength Steel

**DOI:** 10.3390/ma17215314

**Published:** 2024-10-31

**Authors:** Yue Liu, Shun Han, Chao Yang, Ruming Geng, Xiaoyuan Yuan, Yong Li, Chunxu Wang

**Affiliations:** 1 Research Institute of Special Steels, Central Iron and Steel Research Institute Company Limited, Beijing 100081, China; junexplore@163.com (Y.L.); gengruming@nercast.com (R.G.); yuanxiaoyuan@nercast.com (X.Y.); liyong@nercast.com (Y.L.); wangchunxu@nercast.com (C.W.); 2 Department of Materials Technology, AECC Commercial Aviation Engine Company Limited, Shanghai 200241, China; cyang1235@hotmail.co.uk

**Keywords:** ultra-high strength steel, tempering temperature, M_2_C carbides, β-NiAl, synergistic precipitation

## Abstract

The evolution of microstructures and mechanical properties with tempering temperature of a novel 2.5 GPa grade ultra-high strength steel with synergistic precipitation strengthening was investigated. With increasing tempering temperature, the experimental steel initially progressed from ε-carbides to M_3_C and then to M_2_C, followed by further coarsening of the M_2_C carbides and β-NiAl. Concurrently, the martensite matrix gradually decomposed and austenitized. The ultimate tensile strength and yield strength initially increased and subsequently decreased with rising tempering temperature, reaching peak value at 460 and 470 °C, respectively. Conversely, the ductility and toughness initially decreased and then increased with rising tempering temperature, reaching a minimum at 440 °C. The increase in strength was attributed to the secondary hardening effects resulting from carbide evolution and the precipitation of β-NiAl. The subsequent decrease in strength was due to the recovery of martensite and coarsening of precipitates. The decrease in ductility and toughness was linked to the precipitation of M_3_C, while their subsequent increase was primarily attributed to the dissolution of M_3_C and an increase in the volume fraction of reverted austenite. The high dislocation density of martensite, the film of reverted austenite, nanoscale M_2_C carbides, and ultrafine β-NiAl obtained during tempering at 480 °C resulted in the optimal mechanical properties of the experimental steel. The strength contributions from M_2_C carbides and β-NiAl were 1081 and 597 MPa, respectively.

## 1. Introduction

The category of secondary hardening ultra-high strength steel is crucial within the family of ultra-high strength steels (UHSSs) due to its exceptional comprehensive properties, making it extensively utilized in aerospace and other specialized fields [1,2]. Distinguished from low-alloy UHSS that depends on medium carbon martensite matrix strengthening [3] and maraging steel that relies on intermetallic compound strengthening [4], secondary hardening UHSS achieves outstanding mechanical properties through the precipitation of M_2_C (M=Mo, Cr, W) alloy carbides on a medium carbon martensite matrix [5]. The development of secondary hardening UHSS can be traced back to the 1960s in the United States. After over half a century of progress, secondary hardening UHSS has undergone a developmental process from HY180 and AF1410 to AerMet100 and Ferrium M54 [5,6,7,8,9]. Among these representative steel grades, AerMet100 steel stands out because of its optimal balance between strength and toughness while demonstrating exceptional resistance against stress corrosion cracking and fatigue properties. It is employed in advanced aircraft such as the fourth-generation stealth fighter F-22 and carrier-based aircraft F-18A, with tensile strength exceeding 1965 MPa and fracture toughness reaching up to 126 MPa·m^1/2^. The requirements for high-performance structural materials in specialized fields such as aerospace have gradually increased with ongoing development. Although the United States subsequently developed AerMet310 steel and AerMet340 steel, which offer higher strength levels than AerMet100 steel, these advancements still fall short of meeting aerospace demands for new materials. From a microstructure performance perspective, the increase in strength from AerMet100 steel to AerMet340 steel is primarily achieved through the formation of finer and more dispersed M_2_C carbides on a martensite matrix with high dislocation density. However, this strengthening method, which relies solely on the precipitation of M_2_C carbides, leads to an inherent conflict between strength and toughness, creating a performance improvement dilemma for traditional secondary hardening UHSS. To develop a novel secondary hardening UHSS with enhanced strength and greater toughness, numerous scholars have conducted extensive research on synergistic strengthening theories [10]. The β-NiAl phase exhibits remarkable strengthening efficiency and stability due to its lattice structure resembling the martensite matrix. Additionally, it shares an overlapping hardening temperature range with M_2_C carbides. Consequently, researchers have incorporated the β-NiAl phase into traditional secondary hardening UHSS, leading to the development of a novel generation of synergistic strengthening secondary hardening UHSS [11]. The synergistic precipitation mechanism of β-NiAl and M_2_C carbides has been elucidated. Perrut [12] investigated the growth kinetics of these two types of precipitates and found that the growth rate of β-NiAl was significantly lower than that of M_2_C carbides. Although the presence of β-NiAl did not notably affect the growth rate of M_2_C carbides, M_2_C carbides did reduce the growth rate of β-NiAl. Delagnes [13] examined the interaction between these two types of precipitates and revealed that the rapid precipitation of dispersed β-NiAl in the early stages of tempering could provide primary nucleation sites for the precipitation of M_2_C carbides. This process facilitated the formation of more diffuse M_2_C carbides, thereby enhancing the strength of the secondary hardening UHSS.

The precipitation behavior of precipitates is significantly influenced by the tempering temperature compared to tempering time. The tempering temperature not only affects precipitation behavior but also has a substantial impact on the martensite matrix and reverted austenite. Taking AerMet100 as an example [8], the dissolution of the cementite and the precipitation of M_2_C carbides led to an increase in strength as the tempering temperature increased. However, the coarsening of M_2_C carbides and the decrease in dislocation density led to a decrease in strength as the tempering temperature continued to rise. Additionally, the presence of reverted austenite formed during tempering also significantly influences both strength and toughness. A similar phenomenon occurs in secondary hardening ultra-high strength stainless steel. Changes in the characteristics of carbides and reverted austenite due to variations in tempering temperature collectively affect the performance of Ferrium S53 steel [14]. These observations underscore that tempering temperature plays a crucial role in determining the properties of secondary hardening UHSS.

A novel 2.5 GPa secondary hardening UHSS synergistically strengthened by M_2_C carbides and β-NiAl has been developed. The influence of tempering temperature on the microstructure evolution of the experimental steel is more complex compared with traditional secondary hardening UHSS due to the combined strengthening effects of M_2_C carbides and β-NiAl. However, limited studies have been conducted on this aspect in the existing literature. Therefore, this paper aims to elucidate the impact of tempering temperature on the microstructure and mechanical properties of the novel secondary hardening UHSS. The research investigates microstructure evolution and the mechanisms of strengthening and toughening in the experimental steel as influenced by variations in tempering temperature. Emphasis was placed on studying the synergistic strengthening effect of M_2_C carbides and β-NiAl.

## 2. Experimental Methods

### 2.1. Materials Preparation and Heat Treatment

A novel 2.5 GPa secondary hardening UHSS with the nominal chemical composition (wt%) of 0.28% C, 1.5% Cr, 2.5% Mo, 10% Co, 14% Ni, 1.0% Al, and 0.03% Nb was designed based on the notion of synergistic strengthening of M_2_C carbide and β-NiAl. The experimental steel was produced by a vacuum induction melting furnace as 50 kg ingots with a diameter of 100 mm, and then forged into rods with a diameter of 15 mm. All the tested and characterized samples underwent solid-solution treatment at 1020 °C for 1 h, followed by quenching in oil. Subsequently, they were cryogenically treated at −73 °C for 2 h and then heated in air to room temperature, followed by tempering at 300~650 °C for 5 h, and finally cooled in air to room temperature. All samples were named S_300_, S_400_, S_440_, S_460_, S_470_, S_480_, S_490_, S_500_, S_510_, S_520_, S_540_, S_600_, and S_650_ based on their respective tempering temperature.

### 2.2. Mechanical Testing

Tensile tests were performed at room temperature using a tensile testing machine (TTM, GNT50, NCS, Beijing, China) with a strain rate of 0.00025/s. Charpy U-notch (CUN) impact tests were carried out in a Charpy impact-testing machine (CITM, ELYI07, NCS, Beijing, China) at room temperature. The tensile sample size was ϕ3 × 65 mm (gauge length = 15 mm), and the impact sample size was 10 × 10 × 55 mm (notch depth = 2 mm). The data in this paper represent the average of two measurements for each tempering temperature.

### 2.3. Microstructural Characterization

The martensite structure morphology was observed by using an optical microscope (OM, AXIO IMAGER M2m, Zeiss, Oberkochen, Germany). The samples for the OM were ground, polished, and corroded by 4 vol.% Nital for 5~30 s. The morphology of precipitates and reverted austenite was observed using a transmission electron microscope (TEM, FEI Tecnai G2 F20, Thermo Fisher Scientific, Portland, OR, USA) equipped with energy dispersive spectroscopy (EDS). The samples for TEM characterization were polished to a thickness of ~50 μm, and then their central holes were punched via twin-jet electropolishing using a 6 vol.% perchloric acid alcohol solution at −15 °C for about 40 s. The impact fracture morphology was observed using a scanning electron microscope (SEM, EVO 25, Zeiss, Oberkochen, Germany). The volume fraction of reverted austenite was measured by X-ray diffraction (XRD, Co target, D8 Advance, Bruker, Freiburg, Germany), and data collection was conducted at a rate of 2°/min in the 45~115° range at 35 kV and 40 mA. The samples for XRD characterization were electrolytically polished using 10 vol.% chromic acid for 10 s. The calculation equations are based on references [15,16], as follows:

(1) $$V_{\gamma }=1/(1+R_{\gamma }I_{\alpha }/R_{\alpha }I_{\gamma })$$

where *V_γ_* is the volume fraction of austenite, and *I_γ_* and *I_α_* are the integrated intensities of martensite and austenite, respectively. *R_α_* and *R_γ_* are determined by the Miller index of martensite and austenite, respectively, according to reference [15].

## 3. Results and Discussion

### 3.1. Results

#### 3.1.1. Mechanical Properties

**Figure 1:**
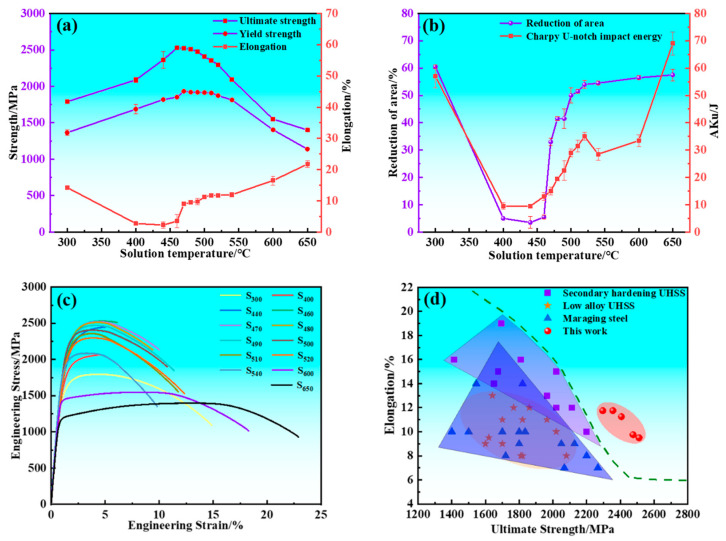
Mechanical properties of samples and comparison with competitive steels. (**a**) Ultimate tensile strength, yield strength, and total elongation; (**b**) Charpy U-notch impact energy and reduction of area; (**c**) Engineering stress–strain curves of samples; (**d**) Tensile properties of samples compared with those of other ultra-high strength steels.

The mechanical properties of the experimental steel are presented in Figure 1. The strength of the experimental steel exhibits a typical secondary hardening behavior with varying tempering temperature. The ultimate tensile strength (UTS) and yield strength (YS) initially increased and subsequently decreased with increasing tempering temperature, reaching peak values of 2526 and 1933 MPa at 460 and 470 °C, respectively. The total elongation (A) and reduction of area (Z) initially decreased and subsequently increased with increasing tempering temperature, and valley values dropped to 2.25% and 3.5% at 440 °C, respectively. The relationship between the U-touch impact energy (Aku) of samples and tempering temperature is divided into four parts for detailed analysis. Within the tempering temperature range of 300~440 °C, the Aku decreased as the tempering temperature rose, and valley values dropped to 9.5 J at 440 °C. The Aku increased with increasing tempering temperature when the experimental steel was tempered in the temperature range of 440~520 °C. The Aku decreased from 35 to 28.5 J when the experimental steel was tempered between 520 and 540 °C. The Aku increased with increasing tempering temperature when the experimental steel was tempered from 540 to 650 °C. S_480_ exhibited an excellent combination of strength, ductility, and toughness. The UTS, YS, A, Z, and Aku were measured as 2511 ± 1 MPa, 1920 ± 5 MPa, 9.5%, 41 ± 0.5%, and 19.5 ± 0.5 J, respectively. As shown in Figure 1c,d, the samples tempered in the temperature range of 480~520 °C demonstrated superior strength and ductility compared to other UHSSs.

**Figure 2:**
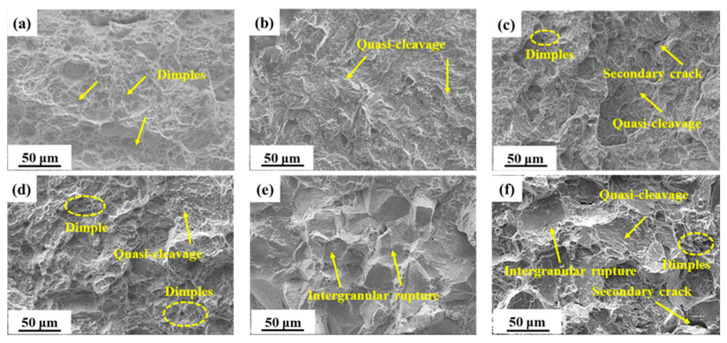
SEM micrographs of impact fractures in samples tempered at: (**a**) 300 °C; (**b**) 440 °C; (**c**) 480 °C; (**d**) 520 °C; (**e**) 540 °C; (**f**) 600 °C.

There is a strong correlation between impact fracture morphology and Aku value, as shown in Figure 2. S_300_, which had a high Aku value, predominantly showed a dimple morphology in its impact fracture morphology. Conversely, S_440_, with a lower Aku value, exhibited mainly a quasi-cleavage morphology. Moreover, S_480_ and S_520_, which both had higher impact values, displayed a combination of dimples and quasi-cleavage in their impact fracture morphology. In the case of S_480_, the presence of secondary cracks was observed. It is noteworthy that S_520_ exhibited a greater number of dimples when compared to S_480_. Not only is the Aku value of S_540_ lower than that of S_520_, but it is also noteworthy that S_540_ exhibited an intergranular fracture morphology that is completely different from the other samples. Lastly, S_600_, which had a higher impact value, exhibited a complex morphology characterized by quasi-cleavage, intergranular rupture, and few dimples.

#### 3.1.2. Microstructure Evolution in Matrix

**Figure 3:**
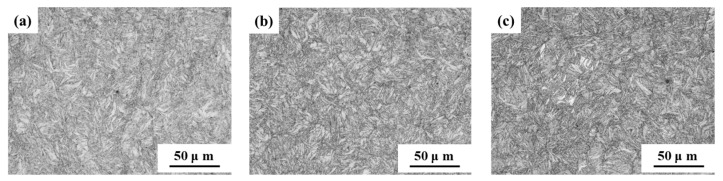

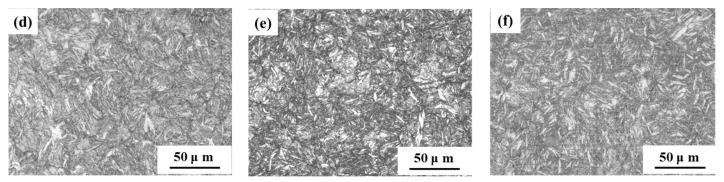
OM micrographs of microstructure in samples tempered at: (**a**) 460 °C; (**b**) 480 °C; (**c**) 500 °C; (**d**) 520 °C; (**e**) 540 °C; (**f**) 600 °C.

The microstructure of the samples is depicted in Figure 3. It was evident that S_460_, S_480_, and S_500_ exhibit a fine and uniform lath martensite microstructure, suggesting minimal decomposition and dislocation recovery of lath martensite. The tempering temperature had little effect on the microstructure of the samples when tempering temperature was below 520 °C. Although the microstructure of S_520_ was also lath martensite, it was observed that the coarsening and decomposition of lath martensite were more obvious than in S_460_, S_480_, and S_500_. This showed that the degree of coarsening and decomposition of martensite gradually increased with the increase of tempering temperature. Specifically, the decomposition and dislocation recovery of lath martensite in S_540_ and S_600_ were particularly pronounced, suggesting that tempering temperatures above 520 °C significantly affect the microstructure of the samples.

**Figure 4:**
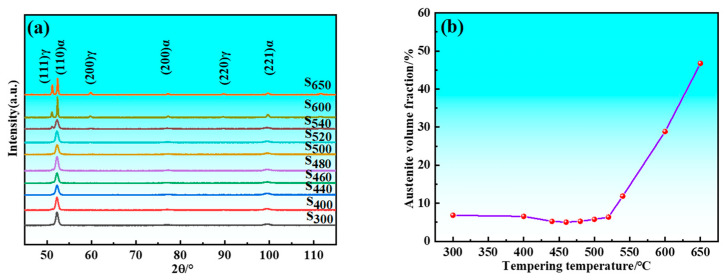
XRD results of samples. (**a**) XRD patterns; (**b**) Austenite volume fraction.

The samples initially retained austenite after quenching, and its volume fraction decreased following cryogenic treatment. There was decomposition of retained austenite and formation of reverted austenite during the tempering process. Figure 4 illustrates the relationship between the total volume fraction of austenite and tempering temperature. Since the only variable among the samples is tempering temperature, Figure 4 still reflects the impact of tempering temperature on the volume fraction of reverted austenite. XRD patterns showed weak austenite diffraction peaks when the tempering temperature was below 520 °C. The intensity of these peaks increased significantly when samples were tempered above 520 °C, indicating a notable increase in the volume fraction of austenite at temperatures exceeding 520 °C. Specifically, the volume fraction of austenite decreased from 6.84% at 300 °C to 5.04% at 460 °C due to greater decomposition of residual austenite than formation of reverted austenite. Conversely, the volume fraction increased from 5.04% at 460 °C to 6.36% at 520 °C because formation of reverted austenite exceeded decomposition of residual austenite. Overall, the volume fraction of austenite showed slight change from 300 to 520 °C, indicating minimal effect of tempering temperature within this temperature range. However, beyond 520 °C, the volume fraction of austenite increased significantly, rising from 6.36% at 520 °C to 46.72% at 650 °C. This demonstrated that the volume fraction of reverted austenite was markedly influenced by tempering temperature at higher levels. It is also evident that the (110)_α_ sharpens with an increase in tempering temperature. This indicates enhanced coherence in the diffraction and reduced dispersion of the diffraction signals. This phenomenon arises from the fact that higher tempering temperatures lead to decreased lattice distortion and dislocation density within the martensite, facilitating the release of stresses and resulting in a more ordered crystal structure.

**Figure 5:**
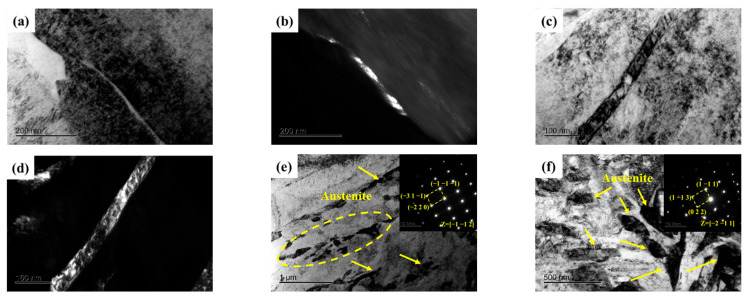
TEM micrographs of reverted austenite in samples. (**a**,**b**) Bright-field and dark-field image of S_480_; (**c**,**d**) Bright-field and dark-field image of S_520_; (**e**) Bright-field image and selected area electron diffraction of S_540_; (**f**) Bright-field image and selected area electron diffraction of S_600_.

**Figure 6:**
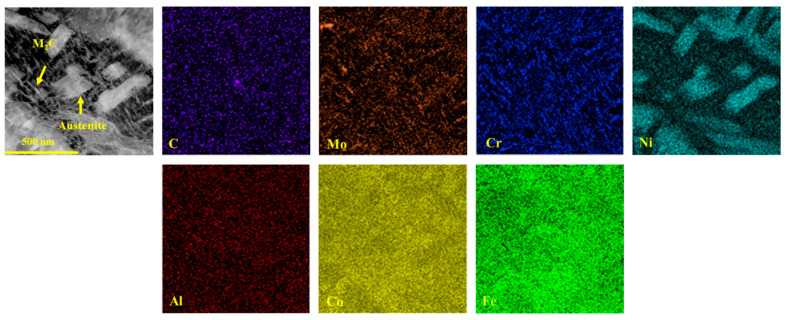
Energy dispersive spectroscopy maps of S_650_.

Not only does the volume fraction of austenite significantly influence the mechanical properties of the samples, but its morphology also plays a critical role in determining these mechanical properties. A noticeable change in the morphology of reverted austenite was observed as the tempering temperature increased, as shown in Figure 5. At lower tempering temperatures of 480 and 520 °C, reverted austenite appeared as a film at the lath boundaries. This film of reverted austenite tended to grow as the tempering temperature increased from 480 and 520 °C. By comparing samples S_520_ and S_540_, it was observed that the film of reverted austenite gradually transformed into massive reverted austenite along the boundaries of lath martensite when the tempering temperature reached 540 °C. There was a significant increase in the size of massive reverted austenite in sample S_600_ compared to S_540_, indicating a gradual growth with increasing tempering temperature. The element maps of S_650_ in Figure 6 reveal the presence of a different phase within the sample. The analysis indicated that the predominant phase was nickel-rich massive reverted austenite. In addition, it could be determined that the phases rich in Al and strong carbide-forming elements (C, Mo, and Cr) were a spherical β-NiAl phase and rod-like M_2_C carbides, respectively, according to a previous article [17].

#### 3.1.3. Precipitate Evolution

The effect of tempering temperature on the precipitates of the samples is complex. On the one hand, the carbides in the samples undergo a nuanced evolution with increasing tempering temperature; on the other hand, the precipitation of carbides is accompanied by the precipitation of intermetallic compounds like β-NiAl. Therefore, this section categorizes tempering temperatures according to two stages (300~460 °C and 460~650 °C) for detailed analysis.

**Figure 7:**
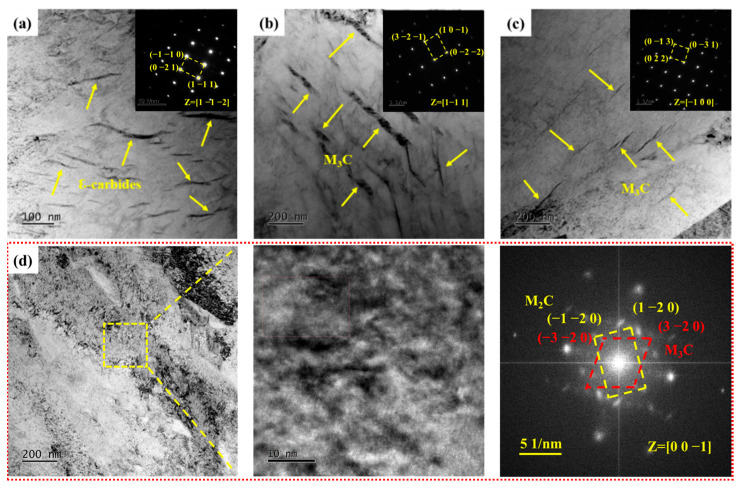
TEM micrographs of carbides in samples. (**a**–**c**) Bright-field image and selected area electron diffraction of S_300_, S_400_, and S_440_, respectively; (**d**) Bright-field image, high-resolution transmission electron microscopy, and fast Fourier transform of S_460_.

The ε-carbides were the primary precipitates when the samples were tempered at 300 °C and were distributed within the martensite matrix as metastable carbides formed during low-temperature tempering. These metastable ε-carbides within the martensite matrix were replaced by more stable lamellar cementite (M_3_C) upon further tempering at 400 °C. There was a significant decrease in both quantity and size of M_3_C compared to S_400_ as the tempering temperature increased to 440 °C, indicating that the M_3_C gradually dissolved into the martensite matrix with the increase of tempering temperature. Figure 7d depicts the TEM topography of the samples after tempering at 460 °C. In Figure 7d, the presence of M_3_C within the field of view is challenging to observe. Based on high-resolution transmission electron microscopy and fast Fourier transform results, it was determined that S_460_ contained two types of carbides: M_3_C and M_2_C carbides. This showed that the dissolution of M_3_C was accompanied by the precipitation of M_2_C carbides when the tempering temperature rose to 460 °C. The dissolution of alloying elements such as C, Mo, and Cr caused by the dissolution of M_3_C provided abundant alloying elements for the formation of M_2_C carbides. In summary, the carbides in the experimental steel evolved sequentially from ε-carbides to M_3_C and finally to M_2_C carbides as the tempering temperature increased from 300 to 460 °C.

**Figure 8:**
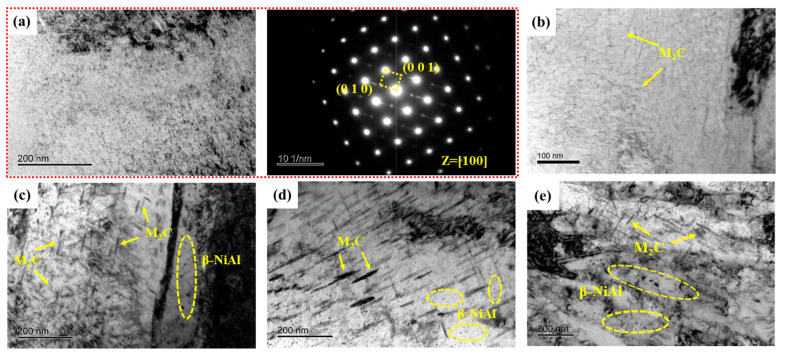
TEM micrographs of precipitates in samples. (**a**) Bright-field image and selected area electron diffraction of S_480_; (**b**–**e**) Bright-field image of S_520_, S_540_, S_600_, and S_650_, respectively.

Figure 8 illustrates TEM photographs of samples tempered at 480~650 °C. Selected area electron diffraction (SAED) results confirm β-NiAl remaining in S_480_. In a previous study [17], 3D-APT results showed a significant element segregation phenomenon in S_480_. There is not only extremely fine β-NiAl but also M_2_C carbides in S_480_. Furthermore, both M_2_C carbides and β-NiAl have a high number density and small equivalent radius. The average equivalent radius and number density of needle-like M_2_C carbides are 2.04 nm and 5.596 × 10^23^ m^−3^, respectively. The average equivalent radius and number density of spherical β-NiAl are 1.66 nm and 1.299 × 10^24^ m^−3^, respectively. Figure 8b illustrates needle-like M_2_C carbides within the martensite matrix after tempering at 520 °C. As tempering temperature increased to 540 °C, the M_2_C carbides transformed from a needle-like to rod-like morphology with increased diameter, accompanied by prominent fine spherical β-NiAl precipitates, as shown in Figure 8c. The diameter and length of the rod-like M_2_C carbides were obviously coarsened when the tempering temperature reached 600 and 650 °C. In contrast, β-NiAl size remained relatively stable compared to the M_2_C carbides across the different tempering temperatures.

### 3.2. Discussion

#### 3.2.1. Effect of Microstructure on Strength

**Figure 9:**
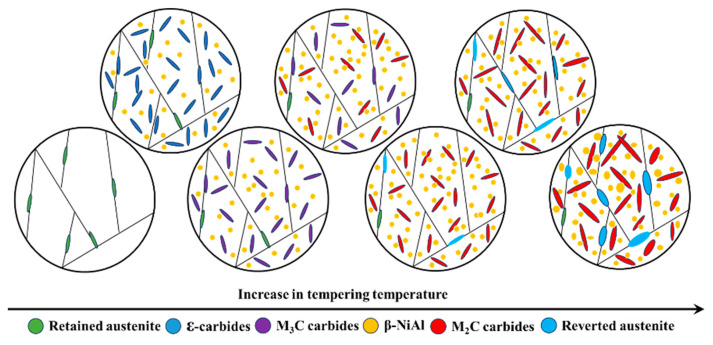
Evolution of microstructures with tempering temperature of the experimental steel.

The results of this study highlighted the significant influence of tempering temperature on both the microstructure and strength of the experimental steel. Moreover, the microstructure underwent a complex evolution as the tempering temperature increased from 300 and 650 °C, as shown in Figure 9. A critical discussion on the relationship between microstructure and strength of the experimental steel is essential. Observing β-NiAl, which is highly coherent with the matrix and has extremely small dimensions, poses a significant challenge. However, although precipitation of β-NiAl could not be directly observed due to constraints, Wang [18] indicated that β-NiAl can co-precipitate with ε-carbides during low-temperature tempering at 200 °C in this type of synergistic strengthening secondary hardening UHSS. Therefore, based on the experimental results of this study, it can be inferred that β-NiAl was present in all samples tempered at various temperatures. As the tempering temperature rose, β-NiAl gradually coarsened, making it easier to observe. The ε-carbides precipitated onto the martensite matrix with high-density dislocations when tempered at 300 °C, playing a role in pinning the dislocations. The martensite matrix with high-density dislocations, ε-carbides, and β-NiAl revealed that the experimental steel possessed a UTS of 1790 MPa and a YS of 1364 MPa at this tempering temperature. The ε-carbides were completely transformed into M_3_C as the tempering temperature rose to 400 °C, resulting in a more pronounced strengthening effect. The M_3_C, β-NiAl, and martensite matrix with high-density dislocations contributed to an increase in the UTS and YS of the experimental steel to 2088 and 1687 MPa, respectively. The M_3_C dissolved gradually with the increase of tempering temperature, providing sufficient alloying elements for the precipitation of M_2_C carbides. This strong secondary hardening effect resulting from M_2_C carbides enabled the experimental steel to reach its peak UTS of 2526 MPa at 460 °C and peak YS of 1933 MPa at 470 °C. The secondary hardening effect of M_2_C carbides is significantly stronger than that of ε-carbides and M_3_C, owing to their more stable chemical composition, crystal structure, and finer size [5,6]. The unapparent change in volume fraction of austenite had little impact on the strength of the experimental steel as the tempering temperature increased from 300 to 460 °C (from 6.84% to 5.04%). Meanwhile, the martensite matrix maintained a high density of dislocations without obvious coarsening and decomposition due to the limited diffusion ability of the alloying elements at lower tempering temperatures. Therefore, the increase in strength of the experimental steel with the increase of tempering temperatures was primarily due to the secondary hardening effect resulting from carbide transformation and the precipitation of β-NiAl.

The microstructure of the experimental steel consisted of a martensite matrix, reverted austenite, M_2_C carbides, and β-NiAl when the tempering temperature increased from 480 to about 650 °C. On the one hand, precipitates grew and coarsened due to the stronger diffusion ability of the alloying elements at higher tempering temperatures. The growth and coarsening of M_2_C carbides and β-NiAl decreased the Orowan strengthening and shear strengthening effects, respectively, leading to the overall weakening of precipitation strengthening of the experimental steel [14]. On the other hand, the martensite matrix gradually coarsened, and the volume fraction of reverted austenite continuously increased with the increase of tempering temperature. The increase in martensite block size and the recovery of dislocations resulting from martensite coarsening diminished the grain boundary strengthening and dislocation strengthening effects of the experimental steel. Additionally, the coarsening of precipitates and the formation of reverted austenite consumed many of the alloying elements such as C, Cr, Mo, and Ni from the matrix. The depletion of these elements in the martensite matrix also diminished the solution strengthening effect of the experimental steel. However, the unapparent change in the volume fraction of austenite did not have a strong effect on strength when the tempering temperature increased from 480 to 520 °C (from 5.27% to 6.36%). In conclusion, the growth of precipitates and the coarsening of martensite were primary factors influencing strength when the experimental steel was tempered within the range of 480~520 °C. The obvious change in the volume fraction of austenite had a strong effect on strength when the tempering temperature increased from 520 to 650 °C (from 6.36% to 46.72%). Therefore, the coarsening of precipitates and austenitization were the principal factors affecting strength when the experimental steel was tempered within the range of 520~650 °C.

Among all the samples, S_480_ exhibited the most excellent comprehensive mechanical properties. The precipitation strengthening mechanism of S_480_ should be thoroughly discussed. There were two types of precipitates after tempering, namely the nanoscale spherical β-NiAl and the needle-like M_2_C carbides, which are different in their manner of precipitation strengthening [19,20,21,22,23].

According to previous studies [24], dislocation shear through β-NiAl produces a shear strengthening effect when the radius of the β-NiAl is less than 2.7 nm due to its coherent structure with the matrix. The shear strengthening is mainly attributed to ordered strengthening, modulus strengthening, and coherent strengthening. Coherent strengthening is the strengthening effect caused by the elastic interaction between the coherent strain field of β-NiAl and the strain field of the dislocations. Coherent strengthening is generally ignored because β-NiAl is highly coherent with the matrix [25]. Therefore, shear strengthening (*σ_Shear_*) is estimated by the following equation:

(2) $$\sigma_{\mathrm{Shear}}=\sigma_{\mathrm{Order}}+\sigma_{\mathrm{Modulus}}$$

Order strengthening (*σ_Order_*) is the strengthening effect caused by the antiphase boundary caused by the dislocations cutting through β-NiAl. Order strengthening (*σ_Order_*) is estimated by the following equations [26]:

(3) σOrder=M4rsf/πTγapb32/b

(4) $$r_{s}=({2/3)}^{1/2}r$$

(5) $$T=G_{\mathrm{Matrix}}b^{2}/2$$

where *M* = 2.8 is the Taylor factor, *b* = 0.248 nm is the Burgers vector of dislocation, *r* = 1.66 nm is the mean radius of β-NiAl, *f* = 2.49% is the volume fraction of β-NiAl, *r_s_* is the average radius of sheared precipitates in the gliding plane, *γ_apb_* = 0.5 J/cm^2^ is the average value of antiphase boundary energy for β-NiAl, *T* is the dislocation line tension, and *G_matrix_* = 80.7 GPa is the shear modulus of the matrix.

Modulus strengthening (*σ_Modulus_*) is the strengthening effect caused by the change in dislocation energy brought about by the dislocations cutting through β-NiAl with a different modulus from the matrix. Modulus strengthening (*σ_Modulus_*) is estimated by the following equations [27]:

(6) $$\sigma_{\mathrm{Modulus}}=M\Delta G\sqrt{3\Delta \mathrm{Grf}}{(0.8-0.143\ln(r/b))}^{2/3}/4\pi^{2}\sqrt{\mathrm{Gb}}$$

(7) $$\Delta G=G_{\mathrm{Matrix}}-G_{\beta -\mathrm{NiAl}}$$

where Δ*G* is the difference between the shear modulus of the matrix and β-NiAl, *G_β-NiAl_* = 77 GPa is the shear modulus of the matrix and β-NiAl.

The dislocations bypassing the M_2_C carbides produces the Orowan strengthening effect in previous studies. Orowan strengthening (*σ_Orowan_*) is calculated using the following equations [28,29]:

(8) $$\sigma_{\mathrm{Orowan}}=2\mathrm{MYKb}\ln(2w_{D}R/b)\sqrt{\ln(2w_{D}R/b)/\ln(2w_{L}R/b}/w_{L}R$$

(9) $$K=G\sqrt{1/0.7}/4\pi$$

(10) $$W_{L}=\sqrt{W_{q}/\varphi }-{2W}_{r}$$

(11) $$1/W_{D}=1/W_{L}+1/W_{r}$$

where *Y* = 0.85, *W_q_* = 0.75, and *W_r_* = 0.82 are the parameters of Orowan dislocation loops, *R* = 2.04 nm is the mean radius of the M_2_C carbides, and *φ* = 2.04% is the volume fraction of the M_2_C carbides.

Therefore, the shear strengthening of β-NiAl and the Orowan strengthening of M_2_C carbides are estimated to be about 597 and 1081 MPa, respectively, according to the above equations.

#### 3.2.2. Effect of Microstructure on Toughness

The evolution of microstructure closely correlated with toughness variation observed in the experimental steel as the tempering temperature increased. The dispersed ε-carbides within the martensite matrix effectively mitigated stress concentration following martensite transformation when tempered at 300 °C [4]. Therefore, the experimental steel exhibited high toughness when tempered at 300 °C. The corresponding impact fracture morphology primarily featured dimples. The brittle M_3_C was prone to becoming the initiation point of cracks [5], leading to a rapid decrease in the toughness of the experimental steel when the tempering temperature rose to 400 °C. Although a part of the M_3_C dissolved after tempering at 440 °C, a significant amount of M_3_C remained on the matrix, continuing to serve as pathways for crack propagation. Therefore, the significant decrease in toughness during the tempering process from 300 to 440 °C was mainly attributed to the presence of a large amount of brittle M_3_C in the experimental steel, which led to a brittle fracture morphology with quasi-cleavage on the impact fracture. The brittle M_3_C was almost entirely replaced by M_2_C carbides when the experimental steel was tempered at 460 °C, leading to an improvement in the toughness of the experimental steel. It is noteworthy that as the tempering temperature increased from 300 to 460 °C, there was decomposition of retained austenite and generation of reverted austenite. Although, decomposition of retained austenite remained predominant, the volume fraction of austenite in the experimental steel was slightly reduced. Therefore, it can be concluded that the reduction in toughness of the experimental steel tempered from 300 to 440 °C was primarily due to the presence of a large amount of M_3_C rather than a decrease in the volume fraction of austenite. The slight increase in toughness of the tempered experimental steel from 440 to 460 °C can mainly be attributed to the dissolution of most of the M_3_C. All the M_3_C carbides in the experimental steel had completely dissolved when the tempering temperature reached 480 °C. The volume fraction of austenite increased, and a film of reverted austenite precipitated from the martensite lath boundary, effectively hindering crack expansion and thereby improving the toughness of the experimental steel. Tempering temperature increased from 480 to 520 °C. On the one hand, the increase in martensite block size resulting from the decomposition of the martensitic matrix reduced the toughness of the experimental steel; on the other hand, the increase in volume fraction of the reverted austenite film in the experimental steel contributed to improving its toughness. Meanwhile, the precipitates showed minimal coarsening and had little effect on the toughness of the experimental steel. Therefore, the increase of toughness of the experimental steel at this stage was due to the increase of the volume fraction of the reverted austenite film. The further increase in tempering temperature resulted in rapid coarsening of the precipitates and the martensitic matrix, which was detrimental to the toughness of the experimental steel. A large number of dislocations accumulated around the large-size precipitates during the process of plastic deformation, and micro-cracks preferentially appeared in these areas [14], reducing the toughness of the experimental steel. Meanwhile, the continuous deformation ability and toughness of the experimental steel were enhanced by the formation of massive reverted austenite. Consequently, the improved toughness of the experimental steel at tempering temperatures exceeding 520 °C was primarily attributed to the formation of abundant reverted austenite. It is noteworthy that the unexpected decrease in toughness observed in S_540_ was due to the partial aggregation of detrimental elements (such as P and S); this is commonly referred to as the second type of temper brittleness, as evidenced by the intergranular impact fracture morphology and in the related literature [30]. The impact fracture morphology still had the characteristics of intergranular fracture when tempered at 600 °C, indicating persistent slight hazards from segregation of harmful elements at grain boundaries. Therefore, although the impact toughness of the experimental steel improved compared with S_540_, it remained lower than that at S_520_.

## 4. Conclusions

1. The experimental steel exhibited an excellent combination of strength, ductility, and toughness after tempering at 480 °C. The UTS, YS, A, Z, and Aku were measured as 2511 ± 1 MPa, 1920 ± 5 MPa, 9.5%, 41 ± 0.5%, and 19.5 ± 0.5 J, respectively. The strengthening of β-NiAl and M_2_C carbides was estimated to be about 597 and 1081 MPa, respectively.
2. Strength initially increased and then decreased with the increase in tempering temperature. The increase in strength was attributed to enhanced secondary hardening resulting from the transformation of carbides from ε-carbides to M_3_C to M_2_C carbides and precipitation of β-NiAl. However, the decrease in strength was due to austenitizing and the coarsening of precipitates.
3. The Aku value initially decreased and then increased with increasing tempering temperature. From 300 to 440 °C, the initial decrease in Aku value was attributed to factors such as the presence of M_3_C. However, when the tempering temperature exceeded 440 °C, the toughness increased due to the formation of reverted austenite and the dissolution of M_3_C. The unexpected decrease in toughness at 540 °C was due to the partial aggregation of harmful elements.

## Data Availability

The original contributions presented in the study are included in the article, further inquiries can be directed to the corresponding author.
